# Immediate and Sustained Improvements in Mood and Stress Associated With Yuna, an AI-Powered Digital Mental Health Intervention: Real-World Retrospective Study

**DOI:** 10.2196/94070

**Published:** 2026-08-25

**Authors:** Kelsey McAlister, Courtney Jewell, Chad Stecher, Jennifer Huberty

**Affiliations:** 1 Fit Minded, Inc., 2901 E Greenway Rd PO Box 30271, Phoenix, AZ, 85046, United States, 1 (602) 935-6986; 2 College of Health Solutions, Arizona State University, Phoenix, AZ, United States

**Keywords:** mental health, digital mental health intervention, real-world evidence, conversational AI, mental health app

## Abstract

**Background:**

AI-powered digital mental health interventions (DMHIs) are a promising approach to address barriers to traditional mental health care. However, real-world evidence of their immediate and sustained benefits remains limited.

**Objective:**

The purpose of this real-world, retrospective study is to explore patterns of perceived mood and stress change associated with the use of Yuna, an AI-powered DMHI. We aimed to (1) describe user demographics and session characteristics, (2) quantify the magnitude of mood and stress change within sessions and over time with continued Yuna use, and (3) identify session-level factors associated with changes in mood and stress.

**Methods:**

Adult Yuna users (aged ≥18 y) who initiated at least one session with the Yuna app were included in this study. Users self-reported mood and stress on Visual Analog Scales (VASs; range 0‐1) before and after sessions. Linear mixed effects models were used to explore the immediate, within-session improvements in mood and stress, and the sustained, between-session changes in symptoms of mood and stress. Linear mixed effects models were also used to examine session-level predictors of within-session improvements, including baseline symptom severity, session duration, total number of unique therapeutic approaches used, safety guardrail activation, and gender.

**Results:**

A total of 5549 real-world users were included (2901/5549, 52.3% female; mean sessions 3.44, SD 9.83). Users demonstrated significant, immediate within-session improvements in both mood (*d*=0.55) and stress (*d*=0.56), with sensitivity analyses yielding consistent results. Between-session analyses revealed gradual improvements in baseline mood (*d*=−0.009) and stress (d=−0.011). Baseline symptom severity was the strongest predictor of immediate, within-session change (mood: β=.118, SE 0.003; *P*<.001; stress: β=.131, SE 0.004; *P*<.001), followed by session duration (mood: β=.027, SE 0.003; *P*=.003; stress: β=.029, SE 0.003; *P*<.001). A greater number of unique therapeutic approaches used was associated with smaller improvements in both outcomes (mood: β=−0.007, SE 0.003; *P*=.043; stress: β=-0.011, SE 0.003; *P*=.002).

**Conclusions:**

Use of Yuna, an AI-powered DMHI, was associated with perceived within-session improvements in mood and stress, with preliminary evidence of gradual improvements in mood and stress across repeated sessions. However, the absence of a control group and potential selection bias preclude causal conclusions. These findings offer promising, real-world evidence for AI-powered DMHIs as accessible, on-demand support tools. Prospective, controlled designs are needed to establish causal effects and evaluate the sustainability of observed improvements.

## Introduction

Low mood (ie, persistent sadness or reduced emotional well-being) and chronic stress represent a major and growing public health challenge worldwide. Depression, a common form of persistent low mood, affects hundreds of millions of individuals globally [1], and emotional stress has steadily worsened across countries and employment groups over the past decade [2]. Together, mood and stress-related concerns represent a major source of disability worldwide, underscoring their substantial individual and societal burden [3]. These concerns are particularly salient in the workplace, where elevated stress, low mood, and emotional exhaustion are linked to diminished productivity, increased absenteeism, and impaired well-being [4-6]. As employers increasingly recognize the impact of both mood and stress on workforce functioning, there is a growing demand for scalable, effective approaches to support employee mental health.

Despite heightened attention, access to timely and effective mental health care remains limited for many working adults. Traditional treatments such as psychotherapy and pharmacological care are often constrained by cost, provider shortages, scheduling barriers, and stigma [7,8]. Employee Assistance Programs (EAPs) are commonly offered as an initial workplace resource, yet usage remains persistently low, in part due to limited awareness, perceived stigma, and short-term or reactive models of care [9,10]. As a result, many employees who experience significant stress or mood symptoms do not receive sustained support through existing workplace systems, highlighting the need for interventions that are both accessible and engaging.

In response to barriers in traditional workplace mental health care, digital mental health interventions (DMHIs) have been increasingly adopted by employers as scalable tools to support employee well-being. These programs typically offer app-based cognitive behavioral strategies, mindfulness content, and stress-management resources [11]. While workplace DMHIs can produce modest improvements in mental health outcomes, real-world effectiveness is often limited by low engagement and high dropout [12-14]. Systematic reviews suggest that DMHIs achieve stronger outcomes and higher adherence when programs include guidance or reminders, raising concerns about the long-term sufficiency of purely self-guided formats and highlighting the potential value of more interactive support beyond self-help tools alone [13,15]. At the same time, stigma, privacy concerns, resource constraints, and scheduling barriers continue to limit uptake of human-delivered services in workplace settings [16-18]. Together, these limitations highlight the need for workplace mental health tools that provide timely, personalized support without requiring traditional appointments.

AI-powered DMHIs represent an emerging approach that may help address these challenges by offering conversational, adaptive support that bridges the gap between unguided self-help apps and traditional human care [19,20]. Conversational AI systems can provide real-time emotional check-ins, coping guidance, and reflective coaching in a format that may feel more engaging and responsive than static digital content, while also reducing barriers related to stigma, scheduling, and discomfort with human interaction [21,22]. One example is Yuna, an AI-powered DMHI, which provides employees with 24/7 access to an AI mental health coach through personalized, on-demand conversations. Yuna has been used by thousands of individuals through workplace partnerships, illustrating its potential as a scalable model for delivering evidence-based support. Despite rapid growth in the availability and adoption of AI-driven mental health solutions, empirical evidence remains limited regarding how these platforms are used in real-world workplace populations and whether employees experience meaningful improvements in mood and stress over repeated engagement.

The purpose of this real-world, retrospective study is to explore the association between use of Yuna, an AI-powered DMHI, and perceived mood and stress change. We aimed to (1) describe user demographics and session characteristics, (2) quantify the magnitude of mood and stress change within sessions and over time with continued Yuna use, and (3) identify session-level factors associated with changes in mood and stress. These findings provide real-world evidence on conversational AI mental health support in workplace settings and help inform the development of scalable AI-powered DMHIs for employees.

## Methods

### Study Design and Participants

This retrospective study analyzed secondary data from Yuna, an AI-powered DMHI. The dataset included self-reported mood and stress and app usage metrics from real-world Yuna users, collected as part of routine product evaluation activities in the Yuna platform. Users were included if they: (1) were adults (aged 18+ y), (2) were located in a country where Yuna is available, and (3) initiated at least one session with the Yuna platform. Eligible participants were identified through a retrospective cohort sampling approach from backend app usage records, spanning from December 29, 2024, to January 12, 2026 (ie, the date the data was extracted). As this study is a retrospective study, no direct recruitment took place. A formal power analysis was not conducted, as the retrospective nature of this study precluded prospective sample size determination. Instead, the analytic sample reflected the full population of eligible users available within the observation window. This sample size was sufficient to support accurate and stable estimation of multilevel model parameters [23]. This study was conducted and reported in accordance with the Strengthening the Reporting of Observational Studies in Epidemiology (STROBE) guidelines for observational cohort studies (Checklist 1).

### Ethical Considerations

The study protocol was reviewed and approved by the Solutions Institutional Review Board (IRB; study ID: 1001) under international expedited review procedures (ie, the study was not deemed exempt) and was conducted in accordance with US human subjects research regulations. The study involved a retrospective analysis of preexisting, deidentified data, with no direct interaction or intervention with users. Investigators did not have access to identifiable private information, and informed consent was waived by the IRB due to minimal risk. User data were collected during routine app use, and users agreed to the platform’s terms and privacy policy, which permit the use of deidentified and/or aggregated data for service improvement and research purposes. The study used data from a global user population, processed in accordance with the platform’s privacy policy. Participants were not compensated in any way for using the app or completing in-app questions or surveys. All data were deidentified prior to analysis.

### App Description and Measures: Yuna App

Yuna is a commercially available mental health and wellness platform offered through workplace partnerships. Yuna’s primary feature is a clinician-informed and evidence-based AI-powered mental health coach that provides users with unlimited, 24/7 support to improve well-being. Yuna incorporates personalization features, such as selecting their preferred voice for the AI mental health coach, to foster a comfortable and individualized interaction. Additional features of Yuna include a library of wellness content, guided tools, and self-help resources. Together, Yuna is an emerging, scalable solution that offers more immediate and accessible support than traditional therapy services.

Yuna’s AI mental health coach draws upon evidence-backed therapeutic frameworks, such as dialectical behavior therapy (DBT), cognitive behavioral therapy (CBT), and acceptance and commitment therapy (ACT). During conversations, the system dynamically applies techniques from these frameworks based on the user’s expressed needs. These techniques are implemented as “therapeutic approaches,” defined as discrete intervention strategies (eg, mindfulness exercises, emotion regulation skills, and cognitive reframing). Yuna leverages over 20 such therapeutic approaches, which are informed by clinician-developed prompts. Therapeutic approach selection follows a dual-layer logic. A central controller model analyzes user input, conversational context, and emotional tone to determine which approach is most appropriate for the user’s presenting needs. Once activated, intraapproach logic guides the delivery of the specific approach sequence to ensure protocol fidelity. All therapeutic approach prompts and clinical protocols were developed by a licensed clinician, and the system undergoes iterative refinement through ongoing clinician review.

**Figure 1.**
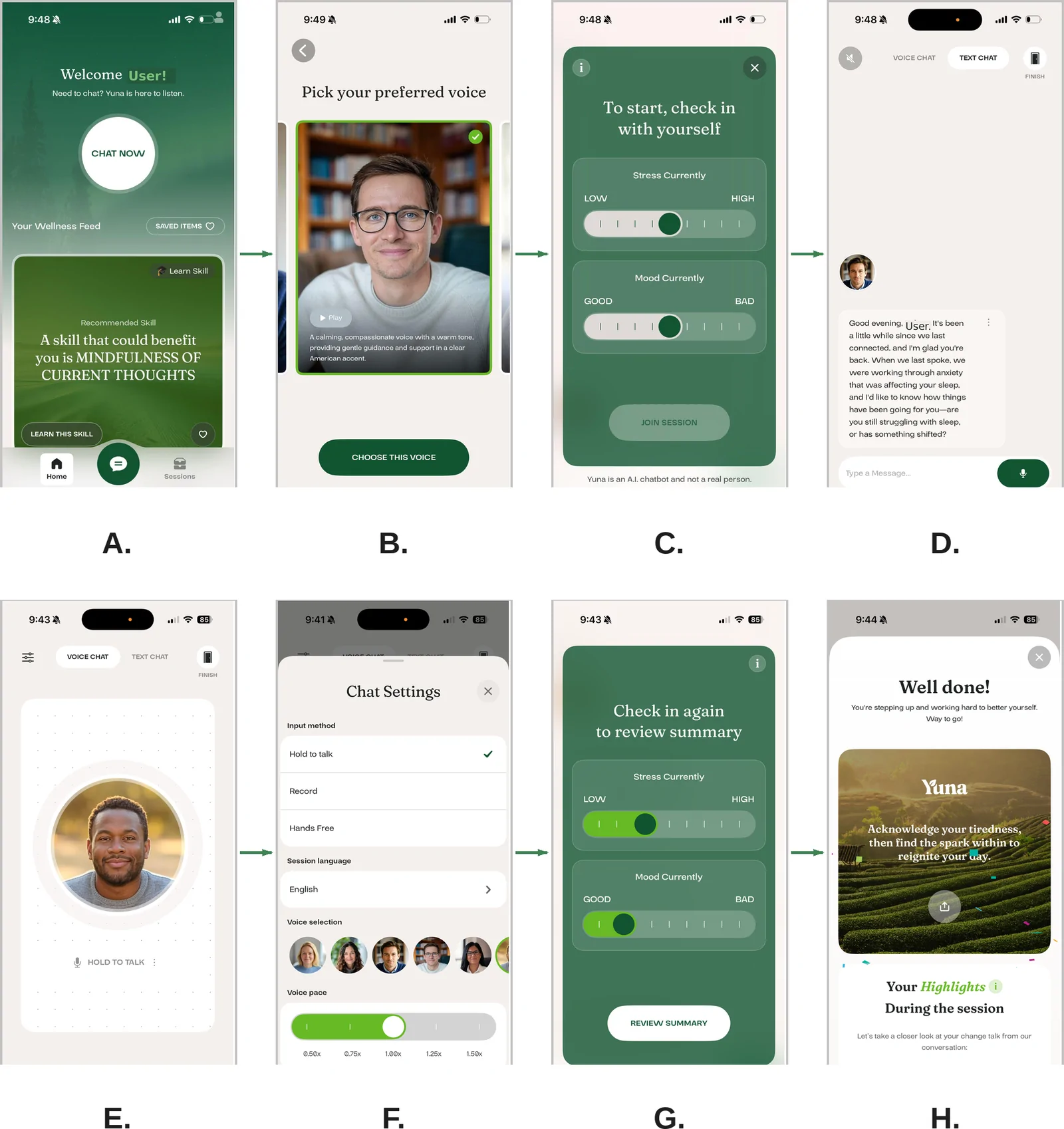
Yuna app user flow across a typical session. Note. (A) home screen with “chat now” entry point. (B) AI coach voice selection during onboarding. (C) Presession mood and stress ratings via sliding scales. (D) In-session text chat interface. (E) In-session voice interaction interface. (F) Chat settings (eg, input mode, language, voice, and pace). (G) Postsession mood and stress ratings. (H) Postsession summary with reflection and highlights.

High-priority safety therapeutic approaches, including suicide risk-specific strategies and safety guardrails, continuously monitor conversations for high-risk content. Upon activation, standard coaching is suspended and users are provided with crisis deescalation support and emergency resources (eg, 988). To mitigate potential bias in AI-generated responses, Yuna’s clinical team periodically audits deidentified session transcripts to identify and address inconsistencies in approach behavior, inappropriate responses, or gaps in protocol adherence. System performance is additionally monitored through automated quality metrics and a human-in-the-loop review process. Figure 1 depicts a standard Yuna user flow.

### Study Measures

#### Overview

When a user first signs up for Yuna, they are able to provide their age and gender within their account profile. Gender was assessed with the item “What’s your gender?” with response options of Male, Female, or Other. User country is derived from backend data.

#### Mood and Stress Levels

In the Yuna app, users are prompted to self-report their current mood and stress levels on a Visual Analog Scale (VAS) immediately before and after a session, defined as a discrete interaction with the AI mental health coach that begins when a user initiates a conversation and ends when the user selects “finish” in the conversation. For stress, users move a slider along a continuum from “Low” to “High,” whereas mood is rated by sliding along a scale from “Good” to “Bad.” The slider-based scale is continuous and does not display numerical values to users. Responses are recorded on a 0‐1 continuous scale to the hundredth decimal place, with 10 tick marks serving as visual anchors to facilitate user responding. This measurement approach allows users to indicate their subjective experiences without being restricted to specific numerical values. Mood and stress ratings before sessions were required, while postsession ratings were optional. This prepost assessment approach is consistent with measurement strategies used in other DMHIs [24].

Single-item VASs minimize user burden and support repeated, in-the-moment assessments within a real-world digital intervention context, and have been used widely in digital health research [25]. However, the measures examined in this study have not been psychometrically validated. These metrics are intended to capture subjective, perceived changes in mood and stress rather than capture clinically validated outcomes.

#### App Engagement

Engagement with the Yuna app was described by users’ time with Yuna, total sessions, and session length. Time with Yuna was defined as the total number of days from a user’s first initiated Yuna session to the last recorded Yuna session, representing the user’s total period of possible exposure to the app, whether or not they were actively engaged daily or monthly. Sessions per user were defined as the total number of sessions each user had completed at the time of data extraction. Session length was defined as the average total minutes each session lasted.

### Statistical Analyses

User demographics and session characteristics were characterized using descriptives, including mean and SD for continuous variables and total count and percentages for categorical variables. Mood and stress scores were scored continuously on a scale from 0 to 1, with higher values representing worse mood or higher stress. Data availability differed by research aim, resulting in separate analytic samples and models for within-session and between-session analyses. Within-session analyses required sessions with complete pre- and postsession ratings of mood and stress. Between-session analyses required users with repeated sessions with presession ratings of mood and stress. Because of this, sample sizes vary across models and are reported with each analysis. This approach aligns with best practices in observational, real-world studies, where complete data across all sessions cannot be assumed [26,27]. To characterize missingness and potential selection effects, missingness was quantified for key variables, and engagement was described using median and IQR for sessions per user. Users included in within-session analyses (ie, users with ≥1 session complete with pre-postsession data) were compared to excluded users. Sessions with complete postsession data were compared to sessions without postsession data. To formally evaluate missingness, a logistic regression model was conducted to predict postsession rating completion from presession mood, stress, gender, repeat-user status, and session number.

To examine within-session improvements in mood and stress, we fit separate linear mixed-effects models (LMMs) predicting mood improvement and stress improvement with a random intercept for the user to account for within-user correlation across repeated sessions. The outcome for the within-session models was a prepost change score computed within each session (ie, presession rating minus postsession rating). From this coding, positive values indicated improvement in mood or stress, whereas negative values reflect worsening mood or stress. To evaluate the robustness of within-session findings, two sensitivity analyses were conducted using the same LMM specifications as the primary model. One sensitivity model restricted the analytic sample to each user’s first completed session to assess whether results were driven by repeat users. The second sensitivity model restricted the analytic sample to users with at least two sessions to evaluate whether findings were driven by one-time or minimally engaged users. To examine between-session improvements in mood and stress, separate LMMs were fit to model presession mood and presession stress over time among users with more than one session. To test whether users began subsequent sessions with improved mood or reduced stress, the outcome was baseline ratings at the start of each session. The fixed effect was the session number, defined as the within-user order of sessions, and the random intercept was the user. In these models, negative coefficients for the session number indicated an improvement, meaning that baseline mood or stress was improved at later sessions relative to earlier sessions. Effect sizes for within-session and between-session changes were calculated as standardized mean differences.

To identify session-level factors that were associated with within-session improvements in mood and stress, we fit fully adjusted LMMs with fixed effects for presession mood or stress, session length, safety guardrail activation, total number of unique therapeutic approaches used (ie, count of distinct therapeutic approaches deployed), and gender, and a random intercept for user to account for correlation across repeated sessions within individuals. Continuous predictors were standardized (z-scored) before modeling to facilitate comparability of coefficient sizes. To contextualize findings related to therapeutic approach use, we conducted an exploratory analysis of the three most frequently used therapeutic approaches: the default module (ie, a general supportive agent that guides the conversation and transitions to specialized agents when deeper support is needed), cognitive defusion (ie, an ACT-based technique that helps users disengage from distressing thoughts), and interpersonal effectiveness (eg, a DBT-based approach focused on assertive communication and boundary setting). Session-level indicators were created for each of these approaches and were included as fixed effects in LMMs predicting whether session improvements in mood and stress.

All models were estimated using restricted maximum likelihood (REML) and were adjusted for gender. *P* values were obtained using Satterthwaite approximation. Given the large number of sessions and users available across all analytic samples, the study was considered adequately powered to detect associations of the magnitude observed. All analyses were conducted in R Version 2024.12.0+4.67 (R Core Team) [28].

### Bias Mitigation

Fit Minded, Inc, uses a standardized bias mitigation protocol across all industry-partnered research, applied prospectively and independent of study outcomes. For this study, the following safeguards were implemented. First, an independent academic researcher (CS), external to the commercial relationship with Yuna, conducted a manuscript-level review encompassing the analysis plan, statistical approach, reported results, and interpretation. This review did not include direct access to the raw dataset or independent reexecution of the statistical models. CS received no financial compensation from Fit Minded, Inc. or Yuna in connection with this study. Second, missingness due to optional pre- and postsession mood and stress ratings was addressed by restricting each analytic model to sessions with complete data for the relevant outcomes and predictors, and additional sensitivity analyses were used to explore how different subsamples may be driving the estimated within-session changes in mood and stress.

## Results

### User Demographics and Session Characteristics

The full data set included 5549 Yuna users (Table 1). Users were predominantly female and primarily located in the United States, with additional representation from more than 100 countries. Users completed 3.44 sessions on average, though this distribution was highly right-skewed (median 1, IQR 1‐2). The mean span between first and last session (ie, time with Yuna) was 29.6 days (median 0 d, IQR 0‐15.9). Approximately 62% (3450/5549) of users completed only one session. Yuna users with ≥2 sessions presented with slightly lower baseline mood and stress scores, on average, compared to single-session users (Multimedia Appendix 1).

Postsession mood and stress ratings were missing for 75.3% (11,985/15,916) of sessions, and more engaged users completed more sessions than users who did not complete postsession ratings (mean 6.66, SD 15.87 vs mean 1.85, SD 3.51, respectively). Full missingness data and comparisons between users and sessions with and without complete data are reported in Multimedia Appendix 1.

**Table 1.** User-level demographics and session descriptives (N=5549 users).

Demographics	Values
Age (years), n (%)	
18‐25	117 (2.1)
26‐35	105 (1.9)
36‐45	44 (0.8)
46‐55	22 (0.4)
55+	10 (0.2)
Missing	5251 (94.6)
Sex	
Female	2901 (52.3)
Male	1996 (36.0)
Missing	652 (11.7)
Country	
United States	3386 (61.0)
India	309 (5.6)
United Kingdom	280 (5.0)
Canada	204 (3.7)
Germany	156 (2.8)
Australia	133 (2.4)
Other^a^	1021 (18.4)
Missing	60 (1.1)
Sessions per user, mean (SD)	3.44 (9.83)
Time with Yuna (days), mean (SD)	29.55 (65.15)
Session length (minutes), mean (SD)	20.38 (21.48)
Presession mood, mean (SD)	0.54 (0.22)
Postsession mood, mean (SD)	0.45 (0.23)
Presession stress, mean (SD)	0.54 (0.24)
Postsession stress, mean (SD)	0.45 (0.24)

a Users from 107 additional countries were collapsed into “Other.” Higher mood and stress values indicate worse mood or higher stress. Lower values indicate better mood or lower stress.

### Within-Session Improvement of Mood and Stress

**Figure 2.**
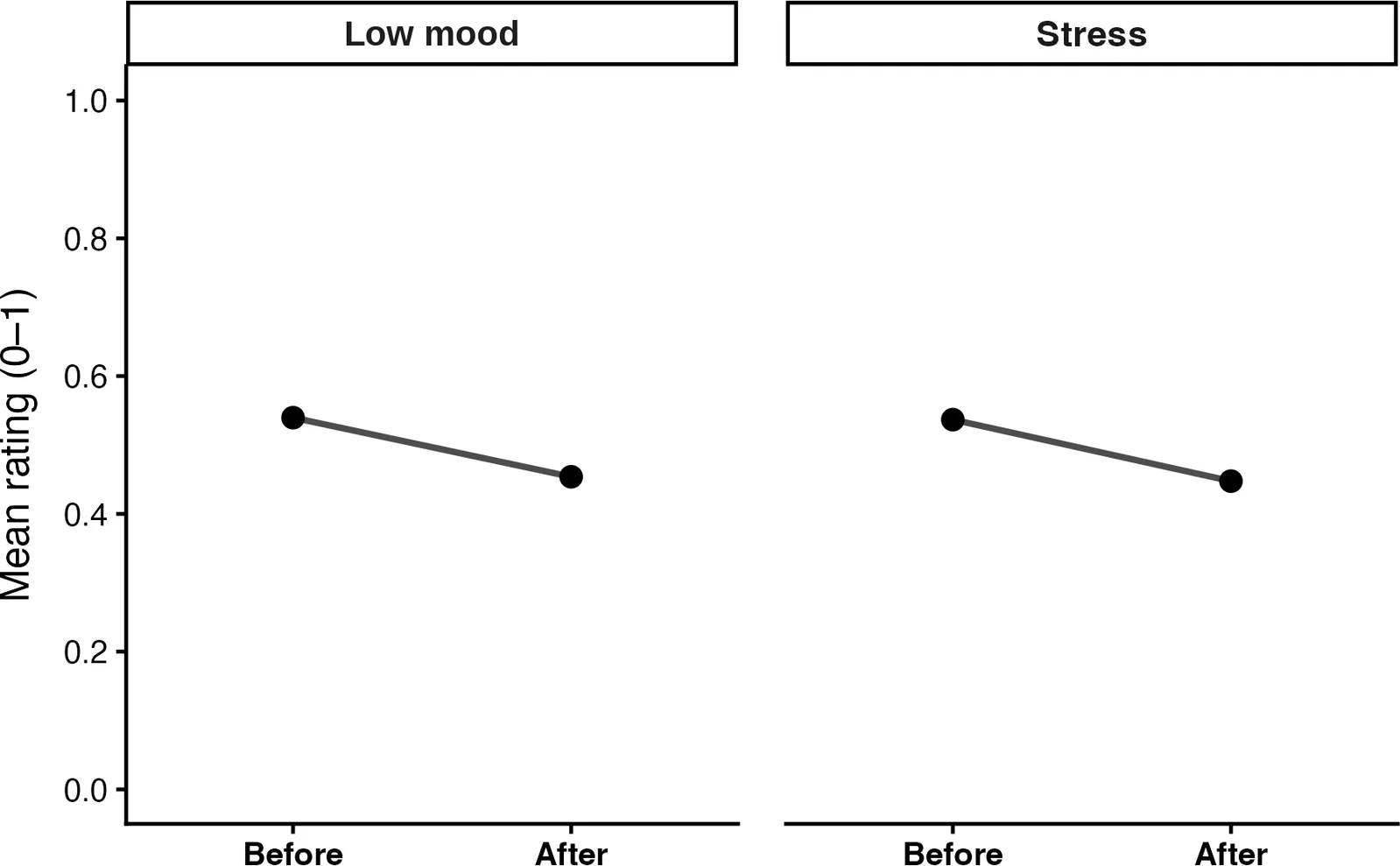
Within-session changes in mood and stress. Mood and stress were assessed using a single-item visual analog scale ranging from 0 to 1, with higher values indicating lower mood and higher stress. Values represent mean prepost session ratings of mood and stress.

LMMs were used to assess the within-session change of mood and stress across participants, adjusted for gender. Yuna users demonstrated statistically significant within-session level improvements in both reported mood (d=0.55) and stress (d=0.56; both *P<*.001). Male users presented with a smaller magnitude of improvements compared to female users, though improvements were positive across groups (Table 2 and Figure 2).

To evaluate the robustness of the within-session findings, two sensitivity analyses were conducted. The first restricted the analytic sample to each user’s first completed session to assess whether primary results were driven by repeat users. The second restricted the samples to users with at least two sessions to assess whether findings were driven by one-time users. Results were consistent with the primary findings, indicating that primary within-session estimates were not driven by the subset of highly engaged users (Multimedia Appendix 1).

**Table 2.** Mixed-effects models of within-session improvement in mood and stress (n=3931 sessions; n=1570 users)^a^.

	Mood				Stress			
	Estimate	SE	95% CI	*P* value	Estimate	SE	95% CI	*P* value
Fixed effects								
Intercept	0.100	0.006	0.09 to 0.11	<.001	0.100	0.007	0.09 to 0.11	<.001
Gender (male)	–0.040	0.009	–0.06 to –0.02	<.001	–0.030	0.010	–0.05 to –0.01	.006
Random effects								
User (intercept), SD	0.110	—^b^	—	—	0.140	—	—	—
Residual, SD	0.180	—	—	—	0.180	—	—	—

a Outcomes represented by within-session level improvement. Positive coefficients indicate improvement in mood or stress. Both models were adjusted for gender. Model diagnostics indicated no singular fits and low multicollinearity. Residual diagnostics suggested some nonnormality and heteroscedasticity, which is common in large observational datasets. For the mood model, adjusted intraclass correlation coefficient (ICC)=0.296, marginal *R*²=0.009, and conditional *R*²=0.302. For the stress model, adjusted ICC=0.357, marginal *R*²=0.004, and conditional *R*²=0.359, indicates not applicable.

b Not applicable.

### Between-Session Changes in Mood and Stress

**Figure 3.**
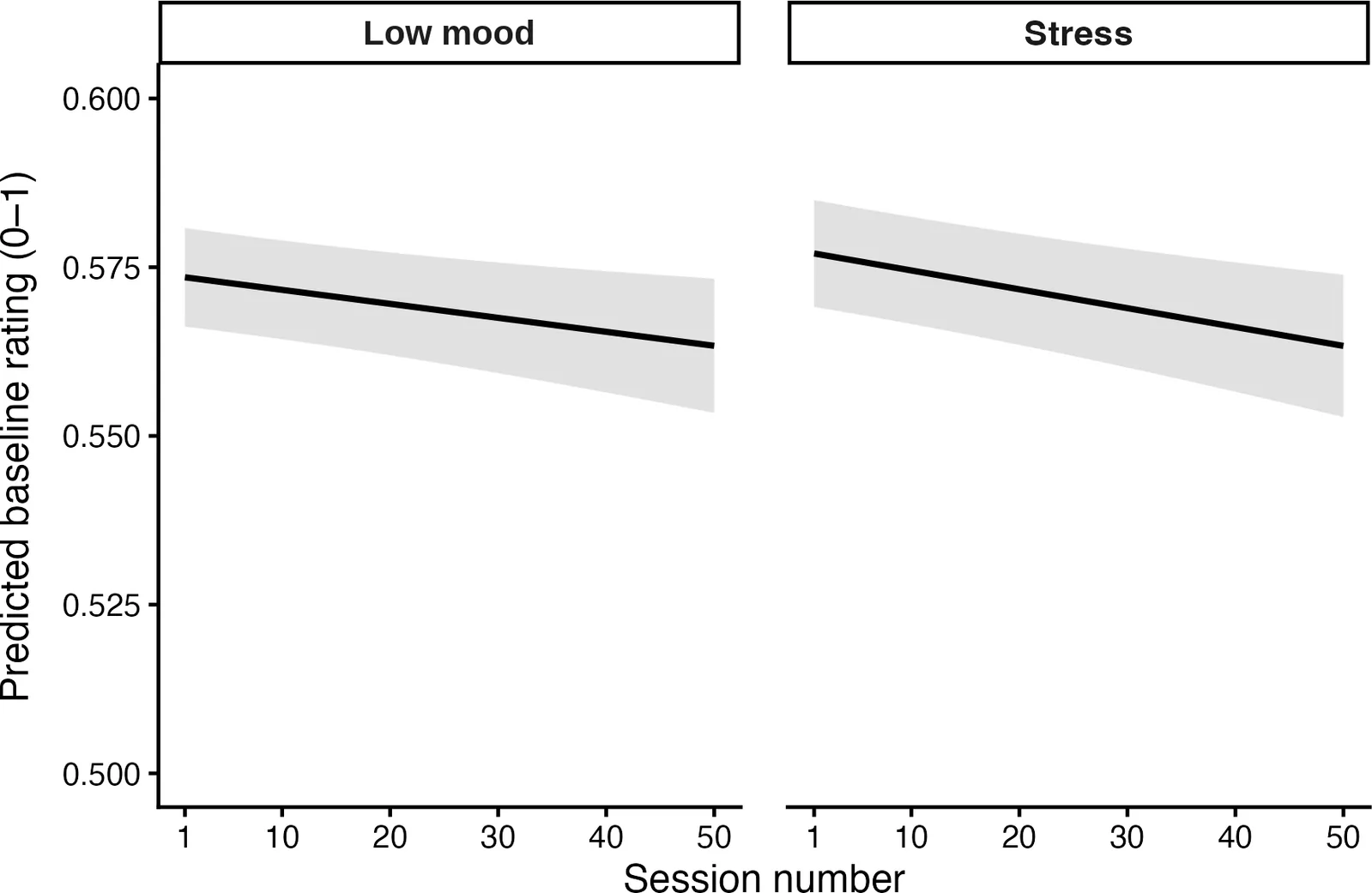
Baseline mood and stress across repeat sessions.

LMMs were used to explore the changes in mood and stress over time. Baseline mood (β=−.00020, SE 0.00008; *P*=.011) and baseline stress (β=−.00029, SE 0.00008; *P*<.001) demonstrated statistically significant decreases across sessions (Table 3 and Figure 3), reflecting gradual improvements in mood (*d*=−0.009) and stress (*d*=−0.011). Gender was a significant predictor of baseline mood, with male users reporting lower presession mood compared to female users (β=−.030, SE 0.008; *P*<.001).

In Figure 3, baseline mood and stress were assessed using a single-item VAS ranging from 0 to 1, with higher values indicating lower mood and higher stress. Predicted baseline mood and stress ratings were estimated from LMMs. Shaded regions represent 95% CIs. The y-axis is truncated to improve the interpretability of between-session trajectories. The full scale ranges from 0 to 1. Truncation does not alter the statistical findings. The small magnitude of observed changes is acknowledged in the discussion.

**Table 3.** Mixed-effects models of between-session change in baseline mood and stress (n=13,877 sessions; n=1838 users)^a^.

	Baseline mood				Baseline stress			
	Estimate	SE	95% CI	*P* value	Estimate	SE	95% CI	*P* value
Fixed effects								
Intercept	0.590	0.005	0.58 to 0.60	<.001	0.590	0.006	0.58 to 0.60	<.001
Session number	–0.00020	0.00008	0.00 to 0.00	.01	–0.00029	0.00008	0.00 to 0.00	<.001
Gender (Male)	–0.030	0.008	–0.04 to –0.01	<.001	–0.020	0.009	–0.04 to 0.00	.03
Random effects								
User (intercept), SD	0.140	—^b^	—	—	0.160	—	—	—
Residual, SD	0.180	—	—	—	0.190	—	—	—

a Baseline mood and stress refer to presession ratings. Session number represents the order of sessions within each user. Negative coefficients indicate lower baseline distress at later sessions. Both models were adjusted for gender. Model diagnostics indicated no singular fits and low multicollinearity. Residual diagnostics suggested some nonnormality and heteroscedasticity. For the mood model, adjusted intraclass correlation coefficient (ICC)=0.378, marginal *R*²=0.004, and conditional *R*²=0.381. For the stress model, adjusted ICC=0.420, marginal *R*²=0.003, and conditional *R*²=0.422.

b Not applicable.

### Session-Level Predictors of Mood and Stress

LMMs were fit to identify session-level factors that were associated with within-session improvements in mood and stress (Table 4). Higher baseline mood (β=.118, SE 0.003) and baseline stress (β=.131, SE 0.004) at the beginning of the session were significantly associated with greater within-session improvement for both outcomes. Longer sessions were significantly associated with greater improvements in mood (β=.027, SE 0.003) and stress (β=.029, SE 0.003), though the magnitude of these coefficients was smaller than the magnitude of baseline stress.

A greater number of unique therapeutic approaches used was associated with smaller within-session improvements in mood (β=−.007, SE 0.003; *P*=.043) and stress (β=–.011, SE 0.003; *P*=.002). Safety guardrail activation showed no significant association with mood or stress improvements. Gender was not significantly associated with within-session improvement after controlling for other session-level factors.

To contextualize findings that a greater number of unique therapeutic approaches were associated with smaller within-session improvements, an exploratory analysis examined outcomes for sessions that included the three most frequently used therapeutic approaches: the default module (17909/42742, 41.9% of sessions), cognitive defusion (3826/42742, 9.0%), and interpersonal effectiveness (3288/42742, 7.7%). In adjusted mixed-effects models including indicators for these approaches, sessions that included the default module were associated with greater improvements in mood (β=.144, SE 0.040; *P*<.001). Cognitive defusion (β=.003, SE 0.007; *P*=.726) and interpersonal effectiveness (β=.008, SE 0.008; *P*=.310) did not reveal significant associations with mood. No therapeutic approach was significantly associated with stress improvements. (Multimedia Appendix 1).

**Table 4.** Session-level predictors of within-session improvements in mood and stress (n=2955 sessions; n=1087 users).^a^

	Mood				Stress			
	Estimate	SE	95% CI	*P* value	Estimate	SE	95% CI	*P* value
Fixed effects								
Intercept	0.106	0.006	0.09 to 0.11	.006	0.108	0.007	0.09 to 0.12	<.001
Baseline symptom severity	0.118	0.003	0.11 to 0.12	<.001	0.131	0.004	0.12 to 0.14	<.001
Session length	0.027	0.003	0.02 to 0.03	.003	0.029	0.003	0.02 to 0.03	<.001
Safety guardrail activation	0.018	0.018	–0.01 to 0.06	.305	0.032	0.018	0.00 to 0.07	.075
Total number of unique therapeutic approaches used	–0.007	0.003	–0.01 to –0.001	.043	–0.011	0.003	–0.02 to –0.01	.002
Gender (male)	–0.018	0.010	–0.04 to 0.00	.083	–0.008	0.011	–0.03 to 0.02	.470
Random effects								
User (intercept), SD	0.119	—^b^	—	—	0.136	—	—	—
Residual, SD	0.140	—	—	—	0.140	—	—	—

a Outcomes represented by within-session level improvement. Positive coefficients indicate improvement in mood or stress. Continuous predictors were standardized (z-scored). Both models were adjusted for gender. Model diagnostics indicated no singular fits and low multicollinearity (variance inflation factors approximately 1.0–2.0). Residual diagnostics suggested some nonnormality and heteroscedasticity, which is common in large observational datasets. For the mood model, adjusted intraclass correlation coefficient (ICC)=0.419, marginal *R*²=0.302, and conditional *R*²=0.594. For the stress model, adjusted ICC=0.483, marginal *R*²=0.323, and conditional *R*²=0.650. - Indicates not applicable.

b Not applicable.

## Discussion

### Principal Findings

The purpose of this real-world, retrospective study is to explore the association between use of Yuna, an AI-powered DMHI, and changes in perceived mood and stress. We aimed to: (1) describe Yuna user demographics and session characteristics, (2) quantify the magnitude of mood and stress change within sessions and over time with continued Yuna use, and (3) identify session-level factors associated with changes in mood and stress. Yuna users were predominantly from the United States (3386/5549, 61.0%) and female (2901/5549, 52.3%). On average, Yuna users spent approximately 30 days with Yuna, with an average of three total sessions. Yuna users showed statistically significant within-session improvements in both mood and stress. Within-session improvements were observed across gender groups, but male users demonstrated slightly smaller improvements compared to female users. Users also demonstrated gradual, between-session improvements associated with Yuna use, entering subsequent sessions with higher baseline mood and lower baseline stress compared to previous sessions. Baseline symptom severity of mood or stress and session duration were associated with greater within-session improvements in mood and stress, whereas the usage of more unique therapeutic approaches (ie, a greater number of distinct therapeutic approach types) was associated with smaller improvements. Safety guardrail activation did not negatively impact improvements in stress or mood, suggesting that safety mechanisms can operate without compromising perceived benefits.

Despite over half of users being from the United States (3386/5549, 61.0%), Yuna demonstrates notable international reach, with users spanning over 110 countries and six continents. This finding highlights the potential of AI-powered DMHIs (like Yuna) to expand access in low- and middle-income countries, where over 75% of individuals do not receive mental health treatment due to limited infrastructure [29]. On average, users engaged with the Yuna platform (ie, time with Yuna) for approximately 30 days, with an average of 3 sessions completed, but demonstrated considerable heterogeneity in their engagement (d with Yuna: mean 29.55, SD 65.15; total number of sessions: mean 3.44, SD 9.83). This suggests that Yuna may be suited to diverse user needs and engagement patterns. Unlike traditional interventions with fixed durations, Yuna’s flexible format aligns with stepped care frameworks that emphasize as-needed support with the capability to modify intensity based on needs and preferences [30,31]. Future research is needed to explore how user engagement and reported outcomes may vary across diverse demographic profiles, especially given some of these tools have international reach [32].

Yuna was associated with within-session mood and stress improvements. Users demonstrated significant improvements in mood and stress following a single, 20-minute session (on average). Within-session effect sizes (*d*=0.55 for mood; *d*=0.56 for stress) are comparable in magnitude to those documented for single-session interventions delivered in controlled therapeutic environments (*g*=0.56 for anxiety; *g*=0.32 overall [33,34]). However, this comparison is illustrative of scale only and does not imply methodological equivalence. Effect sizes from unvalidated, single-item VAS scales cannot be directly compared to standardized mean differences from controlled trials using validated, normed instruments, where regression to the mean, demand characteristics, and selection bias are controlled. Specifically, widely used measures such as the Patient Health Questionnaire-9 and Generalized Anxiety Disorder-7 carry established psychometric properties and clinically defined thresholds for meaningful change that the single-item VAS measures used here do not. The observed within-session improvements demonstrate that Yuna may be associated with immediate benefits through brief, self-initiated conversations with an AI mental health coach, positioning AI-powered DMHIs as a promising option for mood and stress management. A couple of Yuna’s features may explain the observed within-session changes. First, Yuna is an accessible, on-demand platform that connects users with support at moments of high distress and when a sense of support may be most salient [35,36]. Additionally, the judgment-free, user-directed nature of AI-powered DMHIs may influence deeper engagement with the platform’s content. Although male users demonstrated smaller within-session changes compared to female users, they still presented with meaningful improvements. Future research should explore how stigma and gender norms may influence user engagement and outcomes in AI-powered DMHIs. Importantly, whether the within-session improvements observed here reflect transient distress relief or more durable benefit remains an open question. Users may have self-initiated sessions at moments of heightened distress, and the observed improvements may partly reflect natural mood fluctuation following peak distress rather than a lasting change. Future research incorporating follow-up assessments and validated outcome measures would help clarify whether session-level changes translate into meaningful improvements in real-world functioning such as productivity, sleep, or interpersonal relationships.

In addition to immediate symptom relief, AI-powered DMHIs like Yuna may also be associated with sustained, between-session benefits. Users who returned for more than one session showed gradual reductions in baseline stress and mood (ie, lower scores indicating better functioning) across subsequent sessions. However, this finding must be interpreted with considerable caution. The between-session sample represents 33% (1838/5549) of the full analytic sample, and the majority of users completed only a single session and are absent from this analysis. Users who returned are, by definition, a self-selected group most likely to have responded positively to initial use. The observed improvement trend is precisely what survivorship bias would produce, independent of any therapeutic mechanism. These gradual improvements may reflect, but do not confirm, Yuna’s application of therapeutic approaches across sessions. Unlike many AI-generated mental health chatbots that focus on supportive listening or psychoeducation, Yuna leverages therapeutic approaches that are designed to teach evidence-backed skills (eg, cognitive reframing and emotion regulation) and transferable strategies to real-world contexts [37]. If these mechanisms are operative, AI-powered DMHIs may build on previous recommendations and reinforce skills over time, enabling a scaffolded learning experience for users, an approach consistent with prior work indicating that progressive skill-building and reinforcement can enhance skill acquisition [38,39]. However, these interpretations are speculative and are not directly supported by the current data, especially considering the VAS measures used are not validated, so the clinical significance of the observed reductions cannot be confirmed. Lastly, though the between-session coefficients reached significance, their magnitude was small, and the practical significance of these reductions remains uncertain. Future research should incorporate validated outcome measures into routine practice to expand upon these findings and allow for more robust comparisons with traditional intervention modalities.

Several session-level factors were associated with within-session improvements in mood and stress. Baseline symptom severity was the strongest predictor of within-session change: users who entered a session with high baseline stress or lower baseline mood showed significantly greater within-session improvements in both outcomes, suggesting that the association between Yuna use and symptom change is more pronounced when users are experiencing higher levels of distress. This finding aligns with common patterns observed across both traditional interventions and DMHIs [40,41]. However, it is important to acknowledge that baseline symptom severity as the strongest predictor of improvement is also consistent with regression to the mean (eg, [42]). Users may be more likely to initiate sessions during periods of heightened distress, and the observed within-session improvements may partly reflect natural fluctuation in mood and stress rather than a therapeutic response. This alternative explanation cannot be ruled out in the absence of an appropriate control condition.

Longer session durations were positively associated with improvements in both mood and stress, consistent with prior work indicating that increased engagement time in AI-powered DMHIs can support symptom improvement [14]. The number of unique therapeutic approaches activated (ie, clinically-informed strategies and intervention techniques) negatively predicted within-session improvements. In alignment with prior work on intervention complexity, this finding suggests that leveraging a smaller set of focused therapeutic approaches may be more effective in improving baseline mood than integrating numerous therapeutic approaches that may reduce the clarity or impact of individual approaches [43]. Future research should explore and validate how AI-powered DMHIs, such as Yuna, combine, sequence, and select therapeutic approaches during sessions, and how these selections can influence symptom improvement.

Safety guardrail activation was not significantly associated with within-session improvements in mood or stress. This finding suggests that the activation of safety protocols does not detract from the perceived benefits of a session, addressing concerns in the literature about the potential trade-offs between safety mechanisms and engagement with AI-powered DMHIs [44,45]. Well-designed safety guardrails, such as those implemented by Yuna, may not be associated with diminished symptom improvement and may co-occur with stress reduction when discussing a sensitive topic. An important next step will be to qualitatively examine users’ experiences of safety, trust, and disclosure in AI-powered DMHIs to understand how safety guardrail design may influence engagement and symptom improvement.

### Strengths and Limitations

This study has multiple strengths. First, the findings were a combination of self-reported mood and stress measures and app usage metrics from real-world users of Yuna, a commercially available AI-powered DMHI. This approach captures naturalistic user experiences in real-world settings, offering critical insights into outcomes associated with AI-powered DMHI use beyond the controlled conditions of standard efficacy trials [46]. Second, analyses at both the single session and across session levels offered a comprehensive view of the immediate and sustained changes observed across Yuna use. These strengths support the value of real-world evidence for understanding the clinical impact of AI-powered DMHIs, like Yuna, and how these tools function across users and needs.

The study is not without limitations. First, this study does not include a control group, precluding the ability to make causal inferences about the impact of Yuna on mood and stress. Second, demographic data were incomplete, with only 5.4% (298/5549) of users reporting their age range and no collection of other critical variables that may influence response to AI-powered DMHIs, such as education level, socioeconomic status, race, or clinical history. In addition, although gender was assessed directly, the response options offered (Male, Female, and Other) did not follow current best practices for inclusive gender measurement, such as including specific nonbinary or transgender categories or using a two-step gender assessment; this may limit both the precision of gender-based analyses and comparability with studies using more inclusive measures. Though common to retrospective, real-world studies [47], the lack of complete demographic data has direct implications across three domains: (1) without demographic data, we cannot assess whether the analytic sample is representative of the broader population of working adults who might use AI-powered DMHIs; (2) this limits the generalizability of findings to specific age groups, cultural contexts, or clinical populations; and (3) systematic differences between users who provided demographic information and those who did not may introduce bias into observed associations; for example, users who complete profile fields may differ in motivation, digital literacy, or baseline symptom severity. Third, this study included a self-selected sample of Yuna users. Those who are voluntarily engaging in AI-powered DMHIs such as Yuna may differ systematically from the broader population due to differences in motivation or symptom severity. In addition, due to the voluntary nature of app use, data from nonusers for Yuna were not available, and direct comparison between Yuna users and nonusers could not be conducted. As a result, findings should be interpreted with caution and may not generalize to broader populations. Future research should include a prospective study with a larger, more representative sample to understand how effectiveness may vary across demographic groups and profiles, as previous work has reported differences in engagement patterns, therapeutic alliance, and trust with AI-powered DMHIs and AI mental health coaches (like Yuna [32,48]). Fourth, missing data represent a key limitation. Users included in the within-session analyses were more engaged, on average, than those excluded. This means that findings may not generalize to one-time users. While sensitivity analyses among subsamples with different engagement patterns yielded similar results, selection bias may still have influenced the findings. Fifth, the present study relied on a single-item VAS rather than validated outcome measures to assess mood and stress. Although this may influence the reliability and validity of the reported outcomes, it is important to note that these single-item measures are typically used in industry because they enable rapid, low-burden assessments and are more suited for repeated designs [49-51]. Because these single-item scales have not been psychometrically validated, and their reliability and validity for measuring mood and stress have not been formally established, it is not possible to determine whether the observed changes correspond to clinically meaningful improvements. For context, validated instruments such as the Patient Health Questionnaire-9 (PHQ-9) and Generalized Anxiety Disorder-7 Scale (GAD-7) use established thresholds for minimal clinically important differences (eg, a 5-point change on the PHQ-9). The single-item scales used in this study do not permit such comparisons, and findings should be interpreted as reflecting subjective, within-person perceived changes in mood and stress rather than clinically defined symptom change. Future research should integrate repeated validated outcome measures to more accurately capture perceptions of and changes in mood and stress. Future research would benefit from controlled designs to establish causal effects between AI-powered DMHIs relative to standard care. Lastly, the external review conducted by CS constituted a manuscript-level evaluation rather than an independent data audit; the absence of independent verification of the raw data and statistical models is a limitation of the current bias mitigation protocol, and future industry-partnered research should seek to establish more structurally independent oversight at the level of data access and analysis.

### Conclusion

This study found that use of Yuna, an AI-powered DMHI, was associated with both immediate, within-session improvements and gradual, sustained changes in self-reported stress and mood across repeated sessions. Baseline symptom severity was the strongest predictor of within-session improvement, with users entering sessions with higher stress or lower mood presenting with the greatest observed changes, though regression to the mean cannot be ruled out as a contributing explanation. Session duration and the total number of unique therapeutic approaches implemented during the session were also associated with within-session improvements. Safety guardrail activations were not associated with diminished perceived session benefits, suggesting that safety mechanisms can operate without compromising user experience. These findings offer preliminary, real-world evidence that AI-powered DMHIs may represent an accessible and scalable option for mood and stress support. Future research should prioritize prospective, controlled designs to establish causal effects, incorporate validated outcome measures, and explore design features that maximize positive outcomes and user safety.

## Supplementary material

10.2196/94070 Multimedia Appendix 1 Additional tables and data.

10.2196/94070 Checklist 1 STROBE checklist.

## Abbreviations

ACT: Acceptance and Commitment Therapy
CBT: Cognitive Behavioral Therapy
CI: Confidence Interval
DBT: Dialectical Behavior Therapy
DMHI: Digital Mental Health Intervention
EAP: Employee Assistance Program
GAD-7: Generalized Anxiety Disorder-7
ICC: Intraclass Correlation Coefficient
IQR: Interquartile Range
IRB: Institutional Review Board
LMM: Linear Mixed Model
PHQ-9: Patient Health Questionnaire-9
REML: Restricted Maximum Likelihood
SD: Standard Deviation
SE: Standard Error
STROBE: Strengthening the Reporting of Observational Studies in Epidemiology
VAS: Visual Analog Scale
