# Supplementary material for: Immediate and Sustained Improvements in Mood and Stress Associated With Yuna, an AI-Powered Digital Mental Health Intervention: Real-World Retrospective Study
Source: JMIR Mhealth Uhealth. 2026 Aug 25;14:e94070. doi: 10.2196/94070 (PMC13505972; doi:10.2196/94070)
Supplement: Supplementary file 1 [file mhealth-v14-e94070-s001.docx]

**Supplement Material**

**Supplement Material 1. Engagement Groups: Single Sessions versus Repeat Users**

The distribution of sessions per user is shown in Figure S1, illustrating a right-skewed pattern with the majority of users completing only a single session.

Figure S1. Distribution of sessions per user.

Note. Most users completed a single session, with a smaller subset engaging in multiple sessions, resulting in a right-skewed distribution. To assess potential bias from single-session users, all primary analyses were repeated among users with ≥2 sessions, yielding substantively similar results.

To describe heterogeneity in user engagement, single-session users were compared to repeat users on baseline characteristics (Table S2). Approximately 62% of users completed only a single session, while repeat users (≥2 sessions) averaged 7.45 sessions (median = 3). Repeat users presented with slightly lower baseline mood and stress compared to single-session users, though demographic characteristics were similar across groups.

| Table S2. User engagement groups | | |
| --- | --- | --- |
| Characteristic | Single-session users | Repeat users (≥2 sessions) |
| Users | 3,450 | 2,099 |
| Mean sessions | 1.00 | 7.45 |
| Median sessions | 1 | 3 |
| Mean baseline mood | 0.600 | 0.567 |
| Mean baseline stress | 0.616 | 0.573 |
| Female (%) | 51.3% | 53.9% |
| Male (%) | 37.4% | 33.7% |
| Gender missing (%) | 11.3% | 12.4% |

**Supplement Material 2. Missingness, User-Level, and Session-Level Inclusion Comparisons**

To provide transparency regarding data availability across the full dataset, the percentage of missing observations was calculated for all variables (Table S3). Age was missing for 94.6% of users (n = 5,251), and gender for 11.7% (n = 652). Pre-session mood and stress ratings were complete across all sessions, whereas post-session ratings were missing for 75.3% of sessions (n = 14,369), reflecting their optional status within the platform.

| Table S3. Missingness summary. | | |
| --- | --- | --- |
|  | n | % |
| Total sessions (n) | 19,079 |  |
| Total users (n) | 5,549 |  |
| Missing age* | 5,251 | 94.6 |
| Missing gender* | 652 | 11.7 |
| Missing pre-session mood | 0 | 0.0 |
| Missing post-session mood | 14,369 | 75.3 |
| Missing pre-session stress | 0 | 0.0 |
| Missing post-session stress | 14,369 | 75.3 |
| Complete pre/post mood | 4,710 | 24.7 |
| Complete pre/post stress | 4,710 | 24.7 |
| Sessions with complete post data | 4,710 | 24.7 |
| Notes. *calculated at the user-level | | |

To characterize potential selection effects in the within-session analytic sample, users with at least one complete pre/post-session were compared to users with no complete sessions on available characteristics (Table S4). Independent two-sample Welch’s t-tests were used to compare continuous variables, and chi-square tests of independence were used for categorical variables. Included users were significantly more engaged than excluded users (mean sessions: 6.66 vs. 1.85, *t*(1923) = -12.83, *p* < .001), had higher rates of safety guardrail activation (11.4% vs. 3.2%; χ²(1) = 150.85, *p* < .001), and presented with slightly lower initial mood (*t*(3692) = 6.27, *p* < .001) and stress (*t*(3560) = 5.40, *p* < .001) scores. Demographic characteristics were similar across groups.

| Table S4. User-level inclusion comparison. | | | |
| --- | --- | --- | --- |
| User-level characteristic | No complete sessions | ≥1 complete session | p-value |
| Users | 3,714 | 1,835 | - |
| Mean sessions per user | 1.85 | 6.66 | <.001 |
| SD sessions | 3.51 | 15.87 |  |
| Median sessions | 1 | 2 |  |
| IQR sessions | 1 | 5 |  |
| Any guardrail activation | 3.2% | 11.4% | <.001 |
| Mean first baseline mood | 0.601 | 0.559 | <.001 |
| Mean first baseline stress | 0.613 | 0.573 | <.001 |
| Female | 53.4% | 50.0% | 0.43 |
| Male | 36.2% | 35.6% |  |
| Gender missing | 10.4% | 14.4% |  |
| United States | 62.3% | 60.5% | 0.23 |

To evaluate whether sessions with complete post-session data differed systematically from those without, sessions were compared on available session-level characteristics (Table S5). Sessions with complete post-session data showed slightly lower baseline mood and stress, higher proportions of repeat-user sessions, and greater mean therapeutic agent use compared to sessions without post-session data.

| Table S5. Session-level inclusion comparisons  *Sessions with complete post-data v. sessions without post data* | | |
| --- | --- | --- |
| Session-level characteristic | No post data | Post data present |
| Sessions | 14,369 | 4,710 |
| Mean baseline mood | 0.566 | 0.540 |
| Mean baseline stress | 0.562 | 0.537 |
| Guardrail activation | 2.8% | 3.3% |
| Mean log total agents | 2.230 | 2.544 |
| Mean unique agents | 2.151 | 2.310 |
| Repeat-user sessions | 80.7% | 85.5% |
| Female | 57.3% | 51.0% |
| Male | 33.3% | 32.4% |
| Gender missing | 9.5% | 16.5% |

To evaluate whether missingness in post-session ratings was systematic or random, a logistic regression was conducted predicting post-session rating completion from pre-session mood, stress, gender, repeat-user status, and session index (Table S6). Sessions from repeat users were more likely to include post-session ratings, whereas later sessions within a user's sequence were less likely to do so. Baseline mood and gender were also independently associated with completion likelihood.

| Table S6. Logistic regression predicting post-session completion  *Outcome: session includes post-session ratings* | | | |
| --- | --- | --- | --- |
| Predictor | Estimate | SE | p |
| Intercept | -1.224 | 0.067 | <.001 |
| Baseline mood | -0.307 | 0.104 | .003 |
| Baseline stress | -0.187 | 0.097 | .054 |
| Male (vs female) | 0.092 | 0.038 | .015 |
| Gender missing (vs female) | 0.648 | 0.051 | <.001 |
| Repeat user | 0.393 | 0.048 | <.001 |
| Session index | -0.0038 | 0.00064 | <.001 |

**Supplement Material 3. Sensitivity Analysis: First Completed Session Per User**

To test whether within-session effects were driven by highly engaged users with multiple completed sessions, analyses were restricted to each user's first completed session (n = 1,835 users; Table S7). Results were consistent with primary findings. Mean mood improvement was 0.082 (61.6% of sessions improved) and mean stress improvement was 0.096 (62.9% of sessions improved), supporting the robustness of primary within-session estimates.

| Table S7. First completed session per user | |
| --- | --- |
| Users | 1,835 |
| Sessions | 1,835 |
| Mean mood improvement | 0.0817 |
| Median mood improvement | 0.050 |
| Sessions with mood improvement | 61.6% |
| Mean stress improvement | 0.0959 |
| Median stress improvement | 0.060 |
| Sessions with stress improvement | 62.9% |

**Supplement Material 4. Sensitivity Analysis: Repeat Users**

To evaluate whether within-session effects were consistent among users who returned for multiple sessions, analyses were restricted to users with at least two sessions (n = 1,154 users; n = 4,029 sessions; Table S8). Results were consistent with primary findings. Mean within-session mood improvement was 0.088 (62.7% improved) and mean stress improvement was 0.089 (63.1% improved), indicating that primary effects were not attributable to single-session users alone.

| Table S8. Within-session effects across repeat users | |
| --- | --- |
| Sessions | 4,029 |
| Users | 1,154 |
| Mean mood improvement | 0.088 |
| % mood improved | 62.7% |
| Mean stress improvement | 0.089 |
| % stress improved | 63.1% |

**Supplement Material 5. Agent Rankings**

To contextualize findings regarding therapeutic agent use, agents were ranked by frequency of use across sessions, and within-session outcomes were examined descriptively for the three most frequently used agents (Tables S9 and S10). The default module was activated in 41.9% of sessions, followed by cognitive defusion (9.0%) and interpersonal effectiveness (7.7%). Descriptively, sessions involving cognitive defusion and interpersonal effectiveness demonstrated slightly higher mean mood improvements (0.110 and 0.108, respectively) than the default module (0.087), with approximately 67% of sessions showing improvement across both focused agents. In adjusted mixed-effects models, only the default module was significantly associated with mood improvements (Table S11).

| Table S9. Most frequently used therapeutic agents. | | |
| --- | --- | --- |
| Agent | Sessions | % of sessions |
| Default module | 17,909 | 41.9% |
| Cognitive defusion | 3,826 | 9.0% |
| Interpersonal effectiveness | 3,288 | 7.7% |
| Values | 2,101 | 4.9% |
| Reframe module | 1,878 | 4.4% |
| Pros and cons | 1,838 | 4.3% |
| DBT distress tolerance | 1,808 | 4.2% |

.

| Table S10. Within-session outcomes by top agents. | | | | | | |
| --- | --- | --- | --- | --- | --- | --- |
| Agent | Sessions | Users | Mood change | % Improved | Stress change | % Improved |
| Default module | 4,493 | 1,770 | 0.087 | 62.6% | 0.090 | 63.1% |
| Cognitive defusion module | 1,041 | 618 | 0.110 | 67.1% | 0.112 | 67.0% |
| Interpersonal effectiveness | 823 | 509 | 0.108 | 67.6% | 0.100 | 63.7% |

| Table S11. Associations between the top 3 therapeutic approaches and within-session improvement in mood and stress. | | | | | | |
| --- | --- | --- | --- | --- | --- | --- |
| Predictor | Mood Estimate | SE | p | Stress Estimate | SE | p |
| Default module | 0.144 | 0.040 | <.001 | -0.021 | 0.041 | .618 |
| Cognitive defusion | 0.003 | 0.007 | .726 | -0.002 | 0.007 | .814 |
| Interpersonal effectiveness | 0.008 | 0.008 | .310 | -0.008 | 0.008 | .357 |
| Note. Estimates reflect adjusted associations from linear mixed-effects models controlling for baseline symptom severity, session length, safety guardrail activation, gender, and repeated sessions. Positive values indicate greater within-session improvement. | | | | | | |
